# The Relationship Between Menstrual Cycle Irregularities and COVID-19 Vaccination

**DOI:** 10.7759/cureus.49841

**Published:** 2023-12-02

**Authors:** Ala M Aljehani, Shaima A Banjar, Hadil Sultan Alawam, Shams Alowais, Yara Aldraibi, Asayel BinSaif, Glowi Alasiri

**Affiliations:** 1 College of Medicine, Al-Imam Mohammed Ibn Saud Islamic University, Riyadh, SAU; 2 Department of Family Medicine, King Abdulaziz Medical City National Guard Hospital, Riyadh, SAU; 3 Department of Research, Imam Abdulrahman Bin Faisal University, Riyadh, SAU

**Keywords:** menstrual irregularities, dysmenorrhea, vaccines, covid-19, menstrual cycle

## Abstract

Background

After COVID-19 vaccination, females reported irregularities and changes in their menstrual cycle. We aimed to explore the menstrual irregularities following COVID-19 vaccination in Saudi women of childbearing age.

Methodology

The study was a cross-sectional study conducted among women in Riyadh, Saudi Arabia, who had no history of menstrual irregularities before receiving the first dose of the COVID-19 vaccine. The participants filled out an online self-administered questionnaire via Google Form about any menstrual irregularities they experienced after receiving the COVID-19 vaccine.

Results

A total of 535 participants completed the survey. The study found that 41.7% (223) of women experienced menstrual changes after the first dose of the COVID-19 vaccine, increasing to 44.1% (236) after the second dose. The incidence of these changes varied between the first and second doses. For example, the incidence of changes in period duration decreased from 51.6% to 48.3% after the first and second doses, respectively. Similarly, the incidence of delayed periods decreased from 48.4% to 47.9%, while dysmenorrhea increased slightly from 30.9% to 32.2% after the two doses. The incidence of heavier menstrual flow increased from 26.9% to 30.5%, while the incidence of lighter menstrual flow decreased from 26.9% to 24.6% after the first and second doses, respectively.

Conclusions

There is an increased incidence of changes in menstrual cycle after COVID-19 vaccination, particularly in menstrual cycle length, menstrual pain, and the flow of menstruation. Future studies are needed to investigate the potential underlying biological mechanisms.

## Introduction

In early 2020, the Ministry of Health in Saudi Arabia announced the first case of COVID-19 in a citizen who returned from Iran via the Kingdom of Bahrain [1]. To combat this pandemic, Saudi Arabia administered some vaccines against COVID-19, including BNT162b2 and ChAdOx1 [2]. Saudi population showed a good intention to take the COVID-19 vaccine and trusted the health providers [3]. In terms of responses to the first dose of the ChAdOx1 vaccine in Saudi Arabia, no findings were reported regarding post-COVID-19 vaccination infections, hospitalizations, or deaths [4]. This aligns with the declaration of the United States Center for Disease Control and Prevention (CDC) that COVID-19 vaccines are safe and effective to minimize serious illness and decrease hospitalization and mortality [5]. According to meta-analysis, the efficacy of COVID-19 vaccines ranged from 80.2% for mRNA-based vaccines to 94.6% for adenovirus-vectored vaccines based on results from phase 2/3 randomized clinical trials, and higher side effects were more common with the mRNA-based vaccines [6].

Typical adverse effects of vaccines included redness, pain, headache, myalgia, nausea, chills, or fever as documented by the CDC [7]. However, among the unlisted side effects, several recent studies reported menstrual irregularities post-vaccination, suggesting a relationship between vaccination and the menstrual cycle [8-10]. In 2021, the Danish Medicines Agency and the European Medicines Agency received about 2,800 reports describing menstrual disorders after COVID-19 vaccination that generally concerned spontaneous bleeding lasting from one to 14 days or irregular menstrual cycles; however, the Danish Medicines Agency found no causal association between these menstrual cycle bleeding irregularities and COVID-19 vaccination [11], although the CDC encouraged women of reproductive age, pregnant, or breastfeeding to get vaccinated [12]. However, in 2022, a global retrospective cohort study conducted in North America and Europe, which included participants who received any of nine different COVID-19 vaccines, revealed that vaccination is associated with a minor change in the length of the menstrual cycle. This change is likely to be temporary; however, there was no observed alteration in the length of menses [13].

In this study, we aimed to explore the menstrual irregularities following COVID-19 vaccination in Saudi women of childbearing age. The findings of our study can provide doctors with a better understanding of the possible effects of COVID-19 vaccination on the menstrual cycle. This knowledge can enable them to provide more information and advice to their patients regarding any menstrual changes they may experience after getting vaccinated. Additionally, doctors can use this information to help women who rely on their menstrual cycles for family planning purposes by advising them on how to manage any changes that may occur. By doing so, doctors can help their patients make more informed decisions about their health and reproductive choices.

## Materials and methods

Study design and population

This is a cross-sectional study conducted among women in Riyadh, Saudi Arabia. An online self-administered questionnaire via Google Form was designed about associated menstrual irregularities post-COVID-19 vaccination. Participants were asked to fill out the questionnaire on different online platforms, including Twitter, WhatsApp, and Telegram, from February 1, 2022, until May 17, 2022. The inclusion criteria comprised females aged over 17 years, who had experienced menstruation, were currently residing in Riyadh, and did not exhibit changes attributable to other diseases such as hormonal or bleeding disorders. Additionally, participants had received either the first or second dose of the COVID-19 vaccine between December 2020 and October 2021. Informed consent was obtained from all individuals included in this study. Internal Review Board (IRB) approval was obtained from Imam Muhammad Ibn Saud Islamic University (IMSIU) after being reviewed for Ethics in Research on Living Creatures at IMSIU (IRB approval number 231/2022).

The questionnaire

Each participant was asked to fill out a questionnaire consisting of three sections, each containing multiple questions (the first section included eight questions, the second section included four questions, and the third section included eight questions), for a total of 20 questions (Appendices). Only the patients who fulfilled the inclusion criteria would be able to finish until the end.

Statistical analysis

Statistical analyses primarily involved descriptive analysis. We reported the number and frequencies of categorical variables. All statistical analyses were performed using IBM SPSS Statistics for Windows, Version 25 (IBM Corp., Armonk, NY).

## Results

Characteristics of included participants

A total of 793 responded to our survey, and of them, 535 have received the COVID-19 vaccine. More than half of the participants were aged between 17 and 30 years (*n *= 314, 58.7%), 168 (31.4%) participants were aged between 31 and 45 years, and only 53 (9.9%) participants were aged more than 45 years. Approximately 53% (284) of the participants were single, compared to 43% (230) who were married, only 20 (3.7%) participants were divorced and 1 (0.2%) participant was a widow. The majority of the participants (501, 93.6%) were Saudi citizens. Half of the participants reported previous infection of COVID-19 (*n *= 278, 52%). Regarding contraceptive usage and menstrual changes, 13.8% (74) of the participants reported using contraceptives, and 100% (535) reported no changes or menstrual irregularities before receiving the first dose of the COVID-19 vaccine. More than 77% (415) of participants received three doses of vaccine, 20.7% (111) received two doses, and only 1.7% (9) received one dose (Table 1).

**Table 1:** Characteristics of the included participants.

Item	n	%
Age (Years)		
17-30	314	58.7
31-45	168	31.4
>45	53	9.9
Social status		
Divorced	20	3.7
Married	230	43.0
Single	284	53.1
Widow	1	0.2
Nationality		
Resident	34	6.4
Saudi citizen	501	93.6
Have you been infected with COVID-19 before?		
No	257	48.0
Yes	278	52.0
Are you using any kind of contraceptives?		
No	461	86.2
Yes	74	13.8
Did you have any changes or irregularities before the first dose?		
No, it was regular	535	100.0
How many doses did you receive?		
One dose	9	1.7
Two doses	111	20.7
Three doses	415	77.6

Menstrual irregularities and changes

After receiving the first dose, 223 (41.7%) participants reported menstrual changes, of which 67.3% (150) during the first menstruation and 32.7% (73) during the second menstruation after the dose. Table 2 summarizes the features of menstrual irregularities and changes.

**Table 2:** Features of menstrual irregularities and changes. ^a^Other results were adjusted based on the total number of individuals who experienced menstrual changes.

Item	n	%
Have you noticed any menstrual changes after receiving the first dose?		
No	312	58.3
Yes^a^	223	41.7
When did you notice any menstrual changes?		
The first mensuration after receiving the first dose	150	67.3
The second mensuration after receiving the first dose	73	32.7
How many times did the changes appear?		
Only once	93	41.7
2-5 times	110	49.3
6 or more times	20	9.0
Have you noticed any menstrual changes after receiving the second dose?		
No	290	55.1
Yes	236	44.9
When did you notice any menstrual changes?		
The first mensuration after receiving the second dose	137	58
The second mensuration after receiving the second dose	87	37
Third mensuration or more	12	5.1
How many times did the changes appear?		
Only once	75	31.8
2-5 times	117	49.6
6 or more times	44	18.6

**Figure 1:**
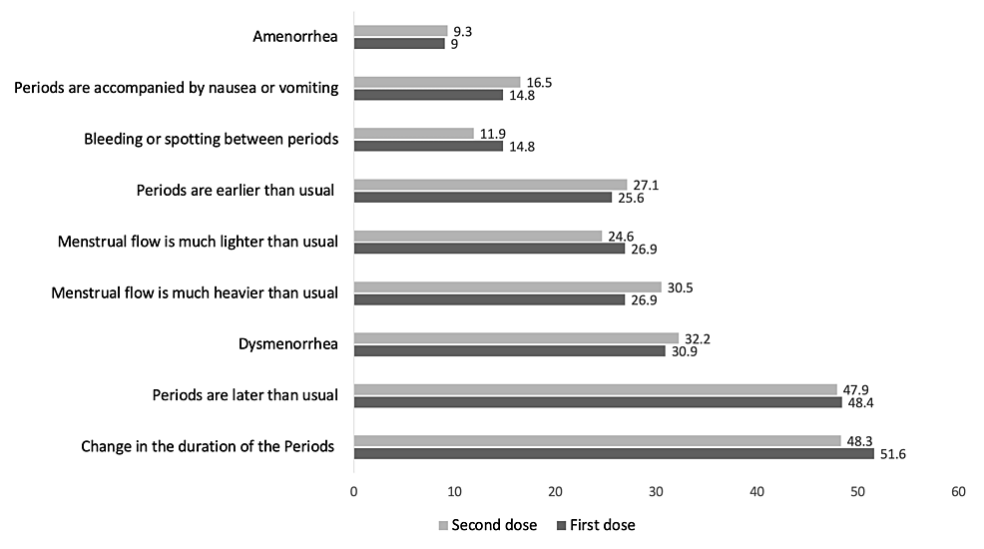
Types of menstrual change after receiving the first and second doses.

These changes occurred two to five times, as reported by 49.3% (110) of those who experienced changes. Additionally, 41.7% (93) reported that these changes happened once, while only 9% (20) reported experiencing them six times or more. Change in the duration of the periods (increased) was the most frequent change reported by 115 (51.6%) participants followed by delayed periods (*n *= 108, 48.4%), then dysmenorrhea (*n *= 69, 30.9%), and heavier/lighter menstrual flow (*n *= 60, 26.9%) (Table 2; Figure 1). 

**Figure 2:**
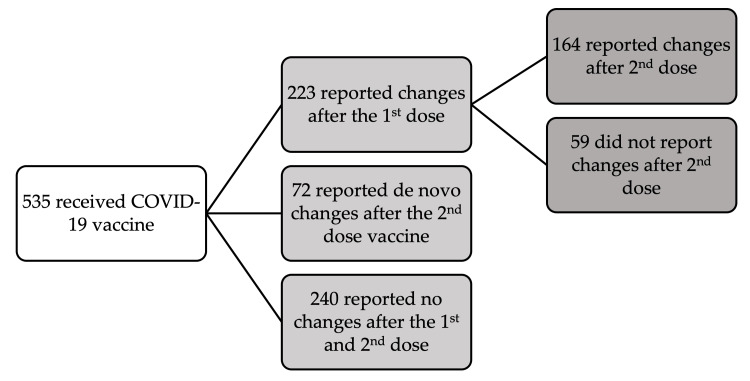
Respondents who received the COVID-19 vaccine and the number of reported changes. Image credit: Hadil Sultan Alawam.

The number of respondents who took the COVID-19 vaccine and the number of reported changes following the first and second doses are represented in Figure 2.

## Discussion

The physiological menstrual cycle length ranges from 26 to 35 days, with menses lasting five days that are initiated with progesterone-responsive decidual cells and executed by prostaglandin E (PGE) and prostaglandin F2-alpha (PGF2α) [14]. However, lifestyle and environmental factors may influence a woman’s menstrual cycle, including, age, weight, stress, exercise, diet, smoking, or air pollution [15]. Since the menstrual cycle is regulated by circulating sex hormones along the hypothalamic-pituitary-gonadal (HPG) axis, upon exposure to stressful stimuli, the hypothalamus and pituitary gland are responsible to secret corticotropin-releasing hormone (CRH) and hence stimulate cortisol hormone synthesis, which, in turn, reduce female sex hormone levels that controls the shedding of the endometrium tissue during menstruation leading to menstrual abnormalities. Also, CRH triggers an inflammatory process through the receptors in female reproductive organs that are involved in ovulation and degradation of the corpus luteum, leading to menstrual abnormalities as well [16].

According to our preliminary findings obtained from 535 participants, the incidence of menstrual changes post-COVID-19 vaccination was 41.7% after the first dose and increased to 44.1% after the second dose in women with no history of changes or menstrual irregularities before receiving the first dose of COVID-19 vaccine. The incidence of increased duration of the period, late period, and lighter menstrual flow was higher in the first dose compared to the second dose; however, the incidence of dysmenorrhea and heavier menstrual flow was higher after the second dose compared to the first dose. Our results are consistent with a cross-sectional study carried out in Saudi Arabia, which found a potential link between the COVID-19 vaccine and menstrual cycle irregularities. After receiving the first dose, the occurrence of a lighter period (24.6%) and a delayed period (27.7%) was higher compared to the second dose. However, After the second dose, there was a higher incidence of a heavier period (17.1%), an earlier period (17.9%), and more severe menstrual cramps (26.8%) compared to the first dose. the type of COVID-19 vaccine did not show any significant associations with the impact on the menstrual cycle [17]. Another study supported our funding and suggested that both COVID-19 vaccination and infection can influence and alter the normal menstrual cycle, resulting in infrequent periods at 25%, frequent periods at 31.53%, irregular periods at 42.93%, prolonged periods at 26.08%, heavy flow at 41.84% at and light flow 20.65% [9]. Further, Lee et al found that 42% of vaccinated participants with regular menstrual cycles experienced heavier menses post-vaccination [18]. Another study found around 25% of vaccinated women reported a change in their menstrual cycle post-COVID-19 vaccination; mainly after the second dose (56%) compared to the first (18%) and third (14%) doses. Among these changes, irregular menstruation was the most common (43%), followed by increased premenstrual symptoms (34%) increased menstrual pain (30%), and heavy bleeding (31%) [8]. Results from the EVA project enrolled 14,153 women showed that 78% of participants reported post-vaccination changes in the menstrual cycle, especially in older women (P <0.001) and smokers (P =0.05), common changes were in terms of menstrual bleeding (43%), pain (41%), delayed menstruation (38%), fewer days of menstrual bleeding (34.5%), and shorter cycle length (32%) [19]. However, our finding percentage was higher than that found in the UK according to the survey conducted in the UK among vaccinated pre-menopausal 4,989 participants that showed a prevalence of menstrual disturbance was 18% up to 4 months after their first dose of the vaccination with increasing the odds in smokers, women with a history of covid-19 disease or participants who are not using estradiol-containing contraceptives [20].

On another hand, several studies have not found strong evidence supporting a significant association between menstrual changes and the COVID-19 vaccine. In a large-scale cohort study conducted in the United States, comparing changes in menstrual cycle or menses length between vaccinated and unvaccinated women, it was found that vaccinated women (with Pfizer-BioNTech vaccine accounting for 55%, Moderna for 35%, or Johnson & Johnson/Janssen vaccine for 7%) experienced a cycle length of less than one day; yet, the unvaccinated participants had no significant changes, which suggested that the COVID-19 vaccination was associated with a minor change in cycle length but not the menses length [21]. Similarly, in a global retrospective cohort study that included nearly 20,000 individuals from Canada, the United Kingdom, the United States, Europe, and other parts of the world who received any of nine different vaccines, the findings confirmed a previous US study. This previous study linked COVID-19 vaccination with a slight increase in menstrual cycle length, less than one day on average. However, this increase did not correlate with any change in the number of days of menses (days of bleeding). Moreover, for the majority of participants in the study, the increase in cycle length resolved in the cycle following vaccination [13].

In another study to investigate the biological and psychological mechanisms that may be responsible for menstrual irregularities during the COVID-19 pandemic, the presence of SARS-CoV-2 IgG antibodies was associated with a higher percentage of menstrual irregularities in unvaccinated women (0% vs. 39%, *P *= 0.026) and increased seven-time risk to menstrual irregularities among women with detectable antibodies compared to women without detectable antibodies (odds ratio 7.03, 95% confident interval 1.39-35.60; *P *= 0.019) [22]. Furthermore, Demir et al. found a positive correlation between the stress/anxiety caused by the COVID-19 pandemic and menstrual cycle dysregulation. The study suggested that the psychological distress caused by the COVID-19 pandemic potentially resulted in changes in the menstrual cycle [23].

Our study also has some limitations. First, the results were based on self-reported data by participants, which may introduce some bias. Second, the findings cannot be generalized to the Saudi population due to the selection of a small sample size and the restriction to one city.

## Conclusions

In conclusion, our findings indicate that, even in the absence of a history of menstrual irregularities, there is an increased incidence of such irregularities following COVID-19 vaccination in Saudi women of childbearing age. The most frequently reported menstrual changes included alterations in the duration of the period, late periods, and dysmenorrhea. Further studies are recommended to investigate the impact of COVID-19 vaccines on women's health and explore the underlying physiological mechanisms.

## Appendices

The first section aims to gather personal details and health history related to menstrual irregularities and COVID-19.

1. Social status: Please indicate your current marital status, choosing one option from Single, Married, Divorced, or Widow.

2. Nationality: What is your nationality? Please select either a Saudi citizen or a resident.

3. Age group: Please specify your age group by selecting one of the following options: 17-30, 31-45, or more than 45

4. COVID-19 infection history: Have you ever been infected with COVID-19 before? Please choose one option: Yes, I have been infected. No, I haven't been infected.

5. Menstrual period: Have you started having regular menstrual periods? Please select one option: Yes, or No.

6. Disease diagnosis: Have you been diagnosed with any medical condition that can cause irregular menstrual cycles? Please choose one option from uterine polyps or fibroids, uterine fibroids, pelvic inflammatory disease, endometriosis, polycystic ovary syndrome, premature ovarian insufficiency, bleeding disorders, hyper/hypothyroidism, or pituitary disorders.

7. Using contraceptives: Are you using any kind of contraceptives? Please select one option: Yes, or No.

8. Menstrual irregularities: Did you have any changes or irregularities before the first dose? Please choose one option: no, it was regular, or yes, it was irregular.

The second section was focused on your COVID-19 vaccination details, including the doses and their dates.

1. Vaccination status: Have you received the COVID-19 vaccination? Please choose one option: Yes, I have received it. No, I haven't received it.

2. Number of vaccine doses: If you have received the COVID-19 vaccination, please indicate the number of vaccine doses you have received from 1 to 3 doses.

3. Date of 1st dose: What month did you receive your first dose? Please choose one option from December 2020, January 2021, February 2021, March 2021, April 2021, May 2021, June 2021, July 2021, August 2021, September 2021, and October 2021.

4. Date of 2nd dose: What month did you receive your second dose? Please choose one option from December 2020, January 2021, February 2021, March 2021, April 2021, May 2021, June 2021, July 2021, August 2021, September 2021, and October 2021.

The third section was to gather information about any menstrual irregularities that may have been experienced after receiving the first and second doses of the COVID-19 vaccination.

1. Menstrual irregularities after the first dose: Have you noticed any menstrual changes after receiving the first dose? Please choose one option: Yes, I have experienced irregularities. No, I haven't experienced any irregularities.

2. Irregularity first shows up after the first dose. If you have experienced menstrual irregularities, please specify when they started to show up. Please choose one option: The first menstruation after receiving the first dose, the second menstruation after receiving the first dose

3. Frequency of irregularities after the first dose: If you have experienced menstrual irregularities, indicate how many times the changes appeared. Please choose one option: only once, 2-5 times, or 6 or more times.

4. Type of irregularities after the first dose: What type of change have you experienced after receiving the first dose? You can choose more than one option: periods are later than usual, periods are earlier than usual, there is a change in the duration of the periods (increased), periods are accompanied by nausea or vomiting, menstrual flow is much heavier than usual, menstrual flow is much lighter than usual, dysmenorrhea (excess pain during periods), amenorrhea (absent periods), bleeding or spotting between periods.

5. Menstrual irregularities after the second dose: Have you noticed any menstrual changes after receiving the second dose? Please choose one option: Yes, I have experienced irregularities. No, I haven't experienced any irregularities.

6. Irregularity first shows up after the second dose. If you have experienced menstrual irregularities, please specify when they started to show up. Please choose one option: The first menstruation after receiving the second dose, the second menstruation after receiving the second dose

7. Frequency of irregularities after the second dose: If you have experienced menstrual irregularities, indicate how many times the changes appeared. Please choose one option: only once, 2-5 times, or 6 or more times.

8. Types of irregularities after the second dose: What type of change have you experienced after receiving the first dose? You can choose more than one option: periods are later than usual, periods are earlier than usual, there is a change in the duration of the periods (increased), periods are accompanied by nausea or vomiting, menstrual flow is much heavier than usual, menstrual flow is much lighter than usual, dysmenorrhea (excess pain during periods), amenorrhea (absent periods), bleeding or spotting between periods.
